# A global dataset of diel activity patterns in insect communities

**DOI:** 10.1038/s41597-024-03408-8

**Published:** 2024-05-30

**Authors:** Mark K. L. Wong, Raphael Didham

**Affiliations:** 1 School of Biological Sciences, The University of Western Australia, Crawley, WA 6009 Australia; 2 Centre for Environment and Life Sciences, Commonwealth Scientific and Industrial Research Organization, Floreat, WA 6014 Australia

**Keywords:** Community ecology, Entomology

## Abstract

Insect activity powers ecosystems and food production globally. Although insect activity is known to vary with the rise and setting of the sun, there is surprisingly limited empirical information on how insect abundance and richness varies across the 24-hour day–night (diel) cycle. Moreover, commonly used methods for sampling insects such as light traps do not provide suitable comparisons of community properties between diel periods. We present a dataset of 1512 observations of abundance and richness during diurnal and nocturnal periods in insect communities worldwide. The data were collected from 99 studies that systematically sampled insect communities during day and night, using sampling methods minimally influenced by diel variation, such as movement-based interception traps. Spanning six continents, 41 countries and 16 insect orders, the data can support investigations into the factors influencing insect diel preferences as well as the causes and consequences of temporal changes in insect biodiversity. The data also provides key baseline information on the diel activity patterns of insect communities for long-term ecological monitoring. These pursuits take on added significance considering contemporary ‘insect declines’ and increasing anthropogenic impacts on diurnal and nocturnal biodiversity.

## Background & Summary

Insects are a major component of biodiversity^1^ and essential to the functioning of ecosystems through their roles in pollination^2^, decomposition^3^, nutrient cycling^4^ and food web dynamics^5^. Despite the tremendous ecological and economic importance of insects, there remains limited knowledge of their temporal activity patterns^6^, especially across the day–night (diel) cycle^7^. Where studied, individual insect species have often displayed distinct diel activity preferences, influenced by factors such as predation risk^8^, climatic fluctuations^9^, resource availability^10^, and evolutionary history^11^. The diurnal and nocturnal preferences of major taxonomic lineages such as bees^12^ and moths^11^ have also been documented. However, it is less clear how diel periods affect the abundance and composition of active insects in ecological communities, and how diel patterns in insect community structure vary across different geographic regions and habitats. A recent meta-analysis revealed extensive variation in the diel activity patterns of insect communities globally, highlighting the potential existence of rich but poorly understood mechanisms^13^.

Documenting diel patterns in the abundance and richness of insect communities can yield insight into fundamental temporal dimensions for species coexistence^14^, and the dynamics of important insect-mediated ecosystem functions^15^. Such work is urgently needed, given growing evidence of insect declines^16^ and the distinct impacts of anthropogenic threats such as climate change^17^ and artificial light pollution^18^ on diurnal and nocturnal communities. Nonetheless, only a limited number of studies have documented patterns in the abundance and richness of insect communities during comparable day and night periods. No standardised, publicly accessible dataset for this basic ecological information also exists. The paucity of data is at least partly due to methodological limitations. Many ‘standard’ sampling techniques for collecting insects are unsuitable for investigations across the entire spectrum of diel activity because they inherently vary in collection efficiency between day and night (e.g., coloured pan-traps during the day, or light traps at night). Other widely used collecting techniques, such as sweep-netting, litter sampling and canopy fogging inadvertently capture inactive individuals.

Here, we present a dataset on the observed diel patterns in abundance and richness in insect communities worldwide. We compiled the data from 99 studies – identified in a literature search – which used methods that exclusively collected active individuals and provided comparable collections across diel periods, such as movement-based interception traps (e.g., pitfall traps, malaise traps, drift nets) and some attraction-based bait traps (e.g., trophic baits) (see Methods).

**Fig. 1:**
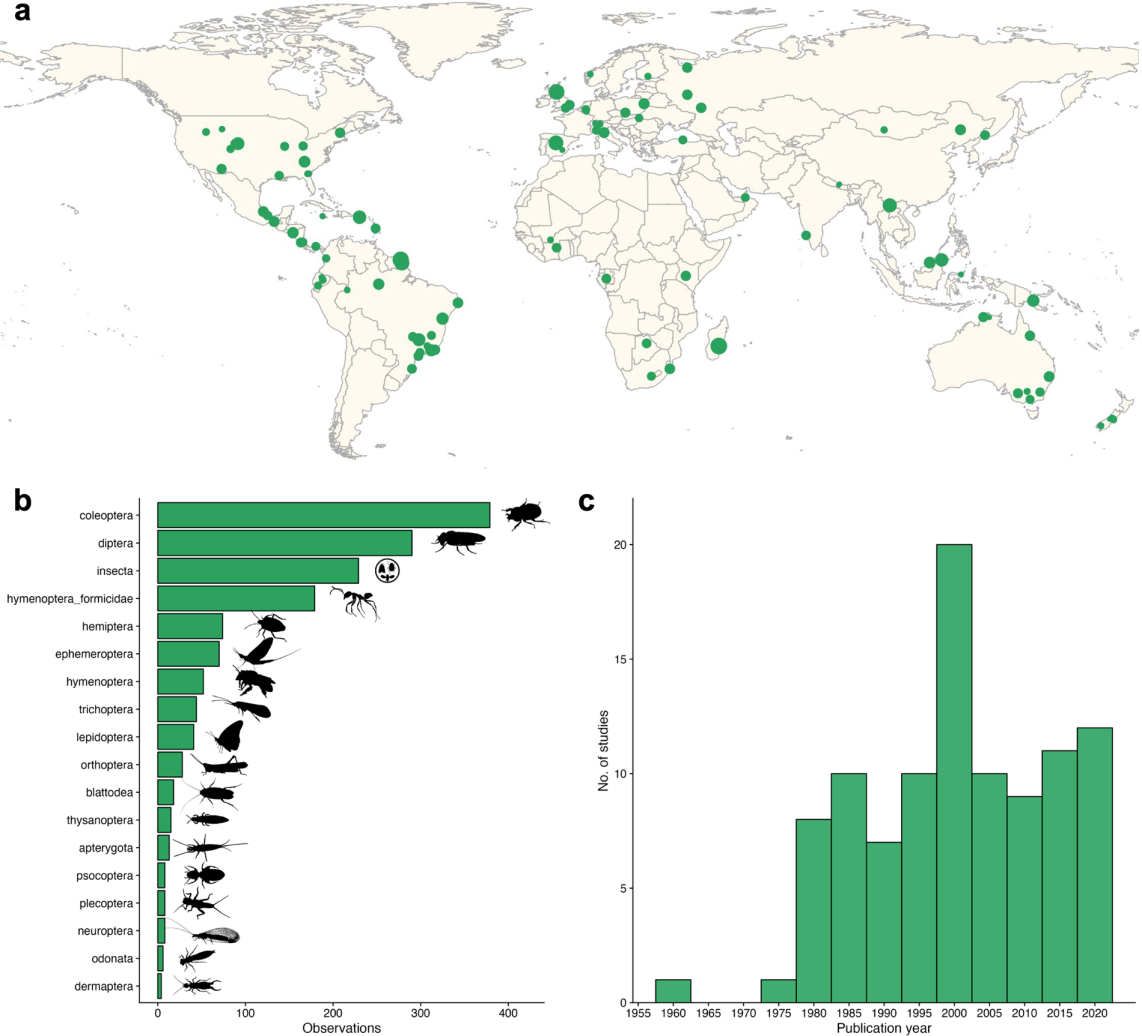
Geographic, taxonomic and temporal coverage of the insect diel activity dataset. (**a**) Global distribution of 123 unique localities where insect community diel patterns were studied. The size of each point corresponds to the relative sampling effort (measured as the number of samples collected) in each observation. (**b**) Distribution of observations in the dataset across 16 insect orders (the additional category ‘insecta’ includes observations of communities comprised of multiple insect orders). (**c**) Distribution of the 99 studies in the dataset across years of publication.

The dataset includes 1512 observations of insect abundance and richness during diurnal and nocturnal periods from 123 unique localities in 41 countries. The observations span all continents except Antarctica (Fig. 1a) and are distributed across tropical and temperate zones in both hemispheres (latitudinal range: 45°S–63°N). Nonetheless, as is symptomatic of global biodiversity data^19^, the observations show some biases in geographic coverage, with notable gaps in Asia and Africa (Fig. 1a).

The taxonomic composition of the insect communities sampled spans 16 insect orders (Fig. 1b). Although most studies sampled communities of flies^20–22^, beetles^23,24^ and ants^25,26^, the dataset nonetheless includes observations for a variety of other insect taxa of diverse trophic levels and functional groups, including taxa that are seldom represented in community studies (e.g., Neuroptera, Hemiptera, Thysanoptera) (Fig. 1b). Still, a substantial number of observations were not identified to order (labelled ‘insecta’ in Fig. 1b), reflecting pervasive taxonomic impediments in ecological studies of insects^27^.

The studies in the dataset span an approximate 70-year period from the 1950s^28^ to the 2020s^29^ (Fig. 1c). Notably, since the 1980s, the number of published studies documenting diel patterns in insect communities during each five-year period has remained relatively constant (ranging between 7–12 studies), except during early the 2000s when these numbers doubled (Fig. 1c). While the overall stable trend reflects the lasting value of information on diel patterns of insect communities, the relatively low numbers underscore that these patterns remain heavily understudied.

Table 1 lists examples of observations in the dataset that collectively illustrate the considerable variation in the diel partitioning of abundance and richness in insect communities. While these records showcase the potentially heterogeneous nature of diel effects on abundance and richness in insect communities, formal meta(analyses) will be required to quantify these effects and elucidate their relationships with abiotic and biotic factors in different habitats and geographic regions. To this end, where available, the dataset also includes values of sample-based means and standard deviations for observations of abundance and richness during specific diel periods. In addition, the dataset includes data for a range of observation-specific environmental variables such as the general ecosystem type (i.e., terrestrial or aquatic), surrounding habitat (e.g., grassland, shrubland, forest, river), vertical habitat stratum sampled (e.g., ground surface, understorey, canopy), and season of sampling (e.g., dry or wet seasons in tropical areas, and cool or warm seasons in temperate areas) based on the information reported in each study. With the geographic coordinates corresponding to each observation, users of the dataset will also be able extract data for other relevant environmental parameters from global databases (e.g., WorldClim^30^).

**Table 1:** Example observations of diel distributions in richness and abundance in insect communities derived from the insect diel activity dataset.

Country	Latitude	Habitat	Taxa	Observed richness			Observed abundance			Study
				*Total*	*D*	*N*	*Total*	*D*	*N*	
Borneo	4.90	forest	Formicidae (ants)	92	82	76	91128	48725	42403	Grevé *et al*.^33^
Brazil	−22.57	forest	Culicidae (mosquitoes)	63	47	39	933	571	362	Alencar *et al*.^34^
Hungary	48.32	river	Chironomidae (midges)	61	55	50	42479	8559	33920	Móra *et al*.^35^
Botswana	−20.45	forest	Scarabaeoidea (dung beetles)	48	14	35	13032	206	12826	Sands *et al*.^29^
Russia	55.45	forest	Carabidae (ground beetles)	14	14	14	1677	797	880	Gryuntal *et al*.^36^
Brazil	−8.07	forest	Calliphoridae (blowflies)	6	6	2	1700	1634	66	Soares *et al*.^22^
USA	43.82	pond	Heteroptera (water bugs)	3	3	3	NA	NA	NA	Hampton & Friedenberg^37^

Presented are observations of seven taxonomic communities from different parts of the world. Observations of richness are in terms of species, while observations of abundance are in terms of individuals. The values correspond to the total number of species or individuals observed across both diel periods (‘Total’), during the day (‘D’), or during the night (‘N’). Note: in addition to the variables presented here, a variety of environmental variables as well as sample-based values for observations are available in the dataset (see main text).

## Methods

We performed a literature search in all Web of Science (WoS) databases (https://webofscience.com) on 28th April 2022 for studies that sampled insect communities across the diel cycle. The search terms used were ‘(insect) AND (community OR communities) AND (activity OR diel OR nocturnal OR diurnal OR night OR day)’. We sorted the results by relevance and screened the abstracts of the first 2000 results to identify relevant publications (beyond the 1350th result, zero to two relevant publications were identified out of every 50 results, while no relevant publications were identified beyond the 1850th result).

We only included studies that systematically sampled insect communities with identical collection methods in day and night periods, which were defined within studies and consistently referred to as the period after sunrise and before sunset, and the period after sunset and before sunrise, respectively. We included studies that used collection methods that collected active individuals, such as movement interception traps (e.g., pitfall traps, sticky traps, malaise traps) and attractive traps (e.g., trophic baits). We excluded studies that used methods which could potentially collect inactive individuals (e.g., sweep-netting, beating) as well as methods for which collection efficiency or attractiveness was influenced by environmental changes across the diel cycle, such as light traps.

For each relevant study, we recorded the total number of different taxa and total abundance of individuals across all day samples, all night samples, and all samples combined. Where reported, we also recorded the mean number of taxa and mean abundance of individuals across all day and all night samples, as well as the corresponding standard deviations (SD) and sample sizes (n). In a few cases where such information was not reported in the text but available from illustrated figures (e.g., bar charts with corresponding error bars), the information was obtained using digital measurements of the figures in ImageJ software^31^. In most studies, each sample was a single collection unit (e.g., a single trap or net). Most studies identified insect taxa to the species level (with a few identifying to the genus, subfamily or family level) and measured insect abundance in terms of the numbers of individuals encountered (a minority used frequencies of occurrence or biomass).

In addition to the observed richness and abundance in insect communities during different diel periods, we recorded information on the geographic location, habitat, sampling period, sampling method and sampled taxa in each study. Using the reported geographic coordinates of the sampled localities, we also determined the surrounding elevation (if not reported within the study) from Google Earth.

## Data Records

A master file is deposited at Figshare^32^. The master file contains:

1. Insect diel activity dataset.
2. Metadata to insect diel activity dataset.

## Technical Validation

All data compilation was performed by the same researcher referring to the same reference material to ensure consistency. To verify the locations of observed insect communities, the reported coordinates of sampling sites were entered into Google Earth and geographic identifiers (e.g., states and road names) were compared to the information reported in each study.

## Usage Notes

The data can contribute to: (i) (meta)analyses on the factors influencing diel patterns in the diversity of insect communities; (ii) knowledge on the diel preferences of different insect taxa and functional groups; (iii) studies on how diel activity patterns of insect communities change over time, through comparisons of the baseline information consolidated here to future observations of insect communities in the same locations.

## Data Availability

No code was used in this study.
